# Evaluating healthcare priority setting at the meso level: A thematic review of empirical literature

**DOI:** 10.12688/wellcomeopenres.13393.2

**Published:** 2018-02-20

**Authors:** Dennis Waithaka, Benjamin Tsofa, Edwine Barasa

**Affiliations:** 1 KEMRI-Wellcome Trust Research Programme, Kilifi, Kenya; 2 Health Economics Research Unit, KEMRI-Wellcome Trust Research Programme, Nairobi, Kenya; 3 Nuffield Department of Medicine, University of Oxford, Oxford, UK

**Keywords:** evaluation, priority setting, resource allocation, meso-level

## Abstract

**Background**: Decentralization of health systems has made sub-national/regional healthcare systems the backbone of healthcare delivery. These regions are tasked with the difficult responsibility of determining healthcare priorities and resource allocation amidst scarce resources. We aimed to review empirical literature that evaluated priority setting practice at the meso (sub-national) level of health systems.

**Methods**: We systematically searched PubMed, ScienceDirect and Google scholar databases and supplemented these with manual searching for relevant studies, based on the reference list of selected papers. We only included empirical studies that described and evaluated, or those that only evaluated priority setting practice at the meso-level. A total of 16 papers were identified from LMICs and HICs. We analyzed data from the selected papers by thematic review.

**Results**: Few studies used systematic priority setting processes, and all but one were from HICs. Both formal and informal criteria are used in priority-setting, however, informal criteria appear to be more perverse in LMICs compared to HICs. The priority setting process at the meso-level is a top-down approach with minimal involvement of the community. Accountability for reasonableness was the most common evaluative framework as it was used in 12 of the 16 studies. Efficiency, reallocation of resources and options for service delivery redesign were the most common outcome measures used to evaluate priority setting.

**Limitations**: Our study was limited by the fact that there are very few empirical studies that have evaluated priority setting at the meso-level and there is likelihood that we did not capture all the studies.

**Conclusions**: Improving priority setting practices at the meso level is crucial to strengthening health systems. This can be achieved through incorporating and adapting systematic priority setting processes and frameworks to the context where used, and making considerations of both process and outcome measures during priority setting and resource allocation.

## Introduction

Priority setting refers to the distribution of resources among competing programmes and patients or patient groups ( Barasa *et al*., 2015b; McKneally *et al*., 1997). Priority setting for health interventions is one of the key challenges facing decision makers worldwide, because resources are scarce, while healthcare needs are unlimited ( Kapiriri *et al*., 2007; Youngkong *et al*., 2009). Priority setting in the health sector occurs at the macro (national), meso (regional, e.g. district/county, or institutional, e.g. hospital), and micro (frontline clinician) level. However, priority setting research has focused on macro and micro level, neglecting meso level priority setting practices ( Barasa *et al*., 2017). Meso level priority setting is crucial given that decentralization has been at the center stage of most health system reforms. Under decentralized systems, regional levels are critical in delivery of healthcare services and control significant resources. In England for example, the primary care trusts (PCTs) were responsible for approximately 80% of the National Health Service budget ( Robinson *et al*., 2012). In Kenya, in the financial year 2016–2017, the counties were responsible for about 60% of the total health sector budget ( Ministry of health, 2017). Further, these regional levels are charged with the daunting task of managing and allocating resources to all public health facilities. How well priorities are set, and how well resources are allocated at the meso/regional level of the health system is therefore a key research and policy question. To contribute to the evidence and knowledge on how well healthcare priorities are set, we conducted a thematic review of empirical literature on meso level priority setting. The objective of the review was to synthesize evidence on findings of studies that evaluated priority setting practices at the meso level in both developed and developing countries.

## Methods

This literature review was broadly guided by the Preferred reporting items for systematic review and meta-analysis protocol (PRISMA) guidelines ( Shamseer *et al*., 2015). However because this is a qualitative thematic review, rather than a quantitative systematic review or meta-analysis, some PRISMA guidance items were not applicable and have been highlighted in the accompanying checklist ( Supplementary File 2).

### Literature search

We searched literature in PubMed, ScienceDirect and Google scholar databases. We used the following search terms: ‘priority setting’ OR ‘healthcare priority setting’ OR ‘resource allocation’ OR ‘healthcare planning’ OR ‘healthcare rationing’ OR ‘budgeting’ OR ‘accountability for reasonableness’ OR ‘program budgeting and marginal analysis’ AND ‘regional health authority’ OR ‘district’ OR ‘meso’ OR ‘county’. We also manually searched reference list of selected papers for other relevant studies.

### Eligibility criteria

We limited the search to studies published in English language that were available from 1997 to 2017. We only included empirical studies that described and evaluated, or those that only evaluated priority setting practice at the meso-level. In this step, we initially screened study abstracts using these criteria and subsequently obtained full-text formats for studies deemed relevant. The final inclusion of studies in the review was based upon a detailed assessment of the full-text formats.

### Characteristics of selected papers

 A total of 1003 papers were found, of which 67 duplicates were removed. Screening by title led to the elimination of 798 articles. This was followed by screening by abstract which led to the elimination of 78 more and finally screening by reading the full papers led to the selection of 16 articles that met the eligibility criteria ( Supplementary File 1).

Table 1 presents a list of the selected papers and their characteristics. Of the 16 studies, both HICs and LMICs had eight each. Five studies were done in Canada, four in Tanzania, three in United Kingdom, two in Kenya, and two in Zambia. Of the 16 studies, 12 sought to describe and evaluate the priority setting process, while four sought to only evaluate the priority setting process. The priority setting activity that was studied across all the selected papers was the planning and resource allocation (or budgeting) process at the regional level. All the studies used a qualitative case study approach. 12 of the studies focused on priority setting across the entire health sector, while four studies focused on priority setting within specific health programmes.

**Table 1.** Characteristics of selected papers.

Study	Country	Study design	Study setting	Priority setting activity	Study objectives	Sector-wide or programme specific
Maluka *et al*., 2010b	Tanzania	Qualitative Case Study	Mbarali District	Planning and allocation of resources (and budget) for essential health service provision in the district	To describes the process of setting healthcare priorities and evaluates the description against accountability for reasonableness	Sector-wide
Maluka *et al*., 2010a	Tanzania	Qualitative Case Study	Mbarali District	Planning and allocation of resources (and budget) for essential health service provision in the district	To explore the acceptability of Accountability for Reasonableness from the perspectives of the Council Health Management Team, local government officials, health workforce and members of user boards and committees.	Sector-wide
Maluka, 2011	Tanzania	Qualitative Case Study	Mbarali District	Planning and allocation of resources (and budget) for essential health service provision in the district	To analyse health care organisation and management systems in Tanzania, and explore the potential and challenges of implementing the AFR approach to priority setting.	Sector-wide
Maluka *et al*., 2011a	Tanzania	Qualitative Case Study	Mbarali District	Planning and allocation of resources (and budget) for essential health service provision in the district	To evaluate the experiences of implementing the AFR approach in Mbarali District, Tanzania using realist evaluation.	Sector-wide
Bukachi *et al*., 2014	Kenya	Qualitative Case Study	Malindi District	Planning and allocation of resources (and budget) for essential health service provision in the district	To describe the healthcare priority setting processes in Malindi district, Kenya, prior to the implementation of AFR in 2008 and evaluates the process for its conformance with the conditions for AFR.	Sector-wide
Nyandieka *et al*., 2015	Kenya	Qualitative Case Study	Malindi District	Planning and allocation of resources	An assessment of priority setting process and its implication on availability of emergency obstetric care services in Malindi District, Kenya (AFR used in assessment)	Program specific
Tuba *et al*., 2010	Zambia	Qualitative Case Study	Kapiri-Mposhi District, Zambia	Planning and resource allocation for malaria services including the distribution of insecticide treated nets.	To describe, evaluate and recommend priority setting process related to malaria services and ITN distribution at the district, facility and community level.	Program specific
Zulu *et al*., 2014	Zambia	Qualitative Case Study	Kapiri-Mposhi District, Zambia	Planning and allocation of resources	To identify local perceptions and practices of fair priority setting (baseline study) as well as at the evolution of such perceptions and practices in priority setting following an AFR based intervention (evaluation study), carried out at district level in Kapiri-Mposhi District in Zambia.	Sector-wide
Mitton *et al*., 2002	Canada	Qualitative Case Study	Chinook Health Region and Calgary Health Region	Resource allocation	To discuss the effectiveness of applying the framework in two regional health authorities in Alberta.	Program specific
Mitton & Donaldson, 2003	Canada	Qualitative Case Study	Calgary Health Region Authority, Chinook Health Region, Headwaters Health Authority in Alberta	Planning and allocation of resources	To evaluate the PBMA framework through survey work and actual case study applications.	Sector wide
Gibson *et al*., 2006	Canada	Qualitative Case Study	Calgary Health Region	Budget planning process and allocation of resources	To use the AFR framework to evaluate the fairness of using PBMA for priority setting and to assess how AFR might make PBMA fairer.	Sector wide
Bravo Vergel & Ferguson, 2006	United Kingdom	Qualitative Case Study	25 Primary care trusts from West Yorkshire and North & East Yorkshire, and Northern lincolnshire	Treatments that offer difficult commissioning choices	To describe and evaluate Primary care trusts with AFR	Program specific
Menon *et al*., 2007	Canada	Qualitative Case Study	7 RHA in Alberta Health region	Planning and allocation of resources	To assess processes for setting health care priorities in Alberta, Canada and whether it is fair using AFR	Sector wide
Robinson *et al*., 2012	United Kingdom	Qualitative Case Study	5 Primary care trusts: Morebeck, Donative, Chatterton, Chetwynd, Nethersole	planning and resource allocation	To investigate local priority-setting activity across five English Primary Care Trusts, between March and November 2012	sector wide
Goodwin & Frew, 2013	United Kingdom	Qualitative Case Study	Plymouth Primary care trusts	Planning and resource allocation	To evaluate PBMA in local healthcare resource allocation	Sector wide
Cornelissen *et al*., 2014	Canada	Qualitative Case Study	Central Okana-gan Local Health Area (LHA)	Resource allocation	To describe and evaluate the process of implementing PBMA in a Canadian regional health authority, and draws out key lessons learned from this experience.	Sector wide

### Quality appraisal

We used the Critical Appraisal Skills Programme (CASP) tool. This entails the use of a check-list approach with screening questions, to assess the reliability, validity and objectivity of the evidence reported in the papers ( CASP UK, 2017; Hannes, 2011). The quality appraisal results are outlined in Table 2.

**Table 2.** Quality appraisal checklist.

Appraisal criteria	Yes	Somewhat	No/Not clear
1. Was there a clear statement of the aims of the research?	16		
2. Is the methodology used for the study appropriate for addressing the research goal?	16		
3. Was the research design appropriate to address the aims of the research? • Has the researcher justified the research design?	16		
4. Is the recruitment strategy appropriate for the study aims? • Researcher explained how the study informants were selected and why these participants were the most appropriate? • Discussion around recruitment i.e. why some people chose not to take part?	12	1	3
5. Was the data collected in a way that addressed the research issue? • If the setting for data collection was justified? • If it is clear how data were collected? • If the researcher has made the methods explicit?	12	2	2
6. Has the relationship between the researcher and the participants been adequately considered? • Researcher reflexivity and potential bias during the formulation of research questions or data collection?	4		12
7. Have ethical issues been taken into consideration? • Informed consent and confidentiality • Approval from ethics committee?	11	1	4
8. Was the data analysis sufficiently rigorous? • In-depth description of the analysis process? • Clarity of the development of themes/categories • Are contradictory data taken into account?	12		4
9. Is there a clear statement of findings? • Explicit findings • Adequate discussion of evidence for and against the researcher arguments • Credibility of finds (triangulation, respondent validation, more than one analyst), findings are discussed in relation to the original research question)	14	2	
10. How valuable is the research? • Researcher discusses the contribution of the study to existing knowledge and understanding • If they identify new areas where research is possible? • If the researchers have discussed whether or how the findings can be transferred to other populations?	16		

The majority of the papers scored poorly in explaining the relationship between the researchers and the participants. We observed that it was not common practice to include the relationship between the researcher and participants under the methods section. Despite this methodological flaw, we found that all the papers provided compelling and valuable insight on the subject matter hence we included all the selected papers in the review.

### Data extraction

We developed a coding framework after reading some of the selected papers, and we used this to develop a coding chart. The coding chart entailed the following; content of priority setting, the process of priority setting and evaluation of priority setting. We then used the coding chart to extract coded data from the selected papers.

### Synthesis of selected papers

We conducted a thematic review of the selected papers. This involved the following steps: (1) reading through the selected papers to identify emerging concepts and ideas, (2) generating a coding framework, (3) reading through the selected papers and coding the contents based on the coding framework (4) charting the coded data, and analyzing by constructing themes from these emergent ideas and concepts in an interpretive stage where findings from the selected papers were integrated into coherent themes. Coding was done manually.

## Results

### Content of priority setting

***Frameworks of priority setting.*** Only seven of the 16 selected studies reported the use of an explicit priority setting framework to guide the priority setting processes. The frameworks were either used independently or in combination. Five papers revealed the use of Program Budgeting and Marginal analysis (PBMA) ( Cornelissen *et al*., 2014; Gibson *et al*., 2006; Goodwin & Frew, 2013; Mitton *et al*., 2002; Mitton & Donaldson, 2003). PBMA is a priority setting framework that involves the retrospective appraisal of resource allocation, broken down into meaningful programmes, with a view to tracking future resource allocation in those same programmes (programme budgeting), and the appraisal of added benefits and added costs when new investment is proposed (marginal analysis), in an incremental way ( Mitton & Donaldson, 2004). One paper revealed the combination of PBMA and multi-criteria decision analysis (MCDA) ( Robinson *et al*., 2012). MCDA ranks healthcare interventions based on scores from a performance matrix that describes the performance of these interventions against a set of agreed upon criteria. One paper revealed the use of a local cultural framework in Zambia ( Zulu *et al*., 2014). This framework employs two cultural principles, ‘ulinganya’ and ‘ukushikwete akapatulula’ to guide decision making. ‘Ulinganya’, in the local Bemba language, means treating people in equal measures while ‘ukushikwete akapatulula’ literally means the absence of prejudice ( Zulu *et al*., 2014).

### Criteria used in priority setting

The reviewed literature reveals the use of various criteria in the priority setting process ( Table 3). The criteria can be broadly classified as either formal or informal criteria. Formal criteria are objective criteria that are used to set priorities. Informal criteria include subjective considerations used in decision making ( Barasa *et al*., 2015b).

**Table 3.** Criteria used to set healthcare priorities in the papers selected for review.

Formal criteria	Number of papers that used	Countries where used
Alignment with national level guidelines and priorities	12	Canada ( Gibson *et al*., 2006; Menon *et al*., 2007; Mitton *et al*., 2002), England ( Bravo Vergel & Ferguson, 2006; Robinson *et al*., 2012) Kenya ( Bukachi *et al*., 2014; Nyandieka *et al*., 2015), Tanzania ( Maluka *et al*., 2010b; Maluka, 2011; Maluka *et al*., 2011a), Zambia ( Tuba *et al*., 2010; Zulu *et al*., 2014)
Economic criteria (Efficiency/cost effectiveness/affordability)	9	Canada ( Cornelissen *et al*., 2014; Gibson *et al*., 2006; Menon *et al*., 2007; Mitton *et al*., 2002; Mitton & Donaldson, 2003), England ( Bravo Vergel & Ferguson, 2006; Robinson *et al*., 2012), Tanzania ( Maluka *et al*., 2010b; Maluka, 2011)
Epidemiological data (burden of diseases and population health indicators)	9	Canada ( Gibson *et al*., 2006; Menon *et al*., 2007; Mitton *et al*., 2002), England ( Goodwin & Frew, 2013; Robinson *et al*., 2012), Kenya ( Bukachi *et al*., 2014) Tanzania ( Maluka *et al*., 2010a; Maluka *et al*., 2010b; Maluka, 2011)
Historical planning and allocation	7	Tanzania ( Maluka *et al*., 2010b; Maluka, 2011; Maluka *et al*., 2011b), Kenya ( Bukachi *et al*., 2014; Nyandieka *et al*., 2015) Canada ( Cornelissen *et al*., 2014; Goodwin & Frew, 2013; Mitton & Donaldson, 2003)
Equity and fairness	6	Canada ( Cornelissen *et al*., 2014; Menon *et al*., 2007), Zambia ( Tuba *et al*., 2010; Zulu *et al*., 2014) Kenya ( Bukachi *et al*., 2014) Tanzania ( Maluka, 2011)
Access	4	Canada ( Cornelissen *et al*., 2014; Gibson *et al*., 2006; Menon *et al*., 2007) Zambia ( Tuba *et al*., 2010)
Wait times	3	Canada ( Cornelissen *et al*., 2014; Menon *et al*., 2007; Mitton *et al*., 2002)
Clinical/population health effectiveness	2	Canada ( Gibson *et al*., 2006) England ( Bravo Vergel & Ferguson, 2006)
Appropriateness	2	Canada ( Gibson *et al*., 2006; Menon *et al*., 2007)
Feasibility	2	Tanzania ( Maluka *et al*., 2010b; Maluka, 2011)
System integration	1	Canada ( Gibson *et al*., 2006)
Informal criteria	Number of papers that used	Countries where used
Political interests	8	Tanzania ( Maluka *et al*., 2010b; Maluka, 2011), Kenya ( Bukachi *et al*., 2014), Zambia ( Tuba *et al*., 2010) Canada ( Gibson *et al*., 2006; Menon *et al*., 2007; Mitton *et al*., 2002), England ( Robinson *et al*., 2012),
Donor and global interests	6	Tanzania ( Maluka *et al*., 2010a; Maluka *et al*., 2010b; Maluka, 2011) , Zambia ( Tuba *et al*., 2010; Zulu *et al*., 2014), Kenya ( Bukachi *et al*., 2014)
Experience/Expertise	6	Tanzania ( Maluka *et al*., 2010b; Maluka, 2011), Kenya ( Bukachi *et al*., 2014) England ( Robinson *et al*., 2012) Canada ( Menon *et al*., 2007; Mitton *et al*., 2002)
Perceptions/interests of regional health managers	4	Tanzania ( Maluka *et al*., 2010b; Maluka, 2011), Zambia ( Tuba *et al*., 2010) Canada ( Gibson *et al*., 2006)

The most common formal criterion used was national level guidelines and priorities. These guidelines were meant to guide regional level priority setting and resource allocation, to ensure they are in line with the national agenda. There was, however, a general consensus that the guidelines limited the ability of regions to set their own priorities ( Bukachi *et al*., 2014; Gibson *et al*., 2006; Maluka *et al*., 2010a; Maluka, 2011; Nyandieka *et al*., 2015; Robinson *et al*., 2012). For example, Nyandieka *et al.* (2015) on assessment of priority setting implications on emergency obstetrics care (EMOC) found that Malindi district in Kenya, had limited freedom in setting priorities as the process is largely dependent on national level guidelines. In Mbarali district in Tanzania, Maluka *et al.* (2010a) found that when district priorities conflicted with national priorities in the planning and budgeting process, the national priorities took precedence. Another common formal criterion used in the studies was efficiency. This criterion was mostly used in HICs, where priority setting frameworks were used as tools for priority setting. For example, in Calgary health region in Canada, cost effectiveness was one of the PBMA criteria used to rank and identify areas for resource reallocations amongst clinical services during the budget-planning process ( Gibson *et al*., 2006). However, efficiency criterion was not always successfully used. Robinson *et al.* (2012) observed that during the planning and priority setting process, attempts to withdraw or reduce services and technologies that offered little health benefit relative to their cost in four primary care trusts in England, were hampered by lack of evidence and culture of resistance to change. One of the reasons identified in LMICs that contributed to the relatively less use of efficiency criterion in priority setting was the lack of quality data. For example, In Mbarali district in Tanzania, the use of cost effectiveness evidence tended to be a small component of the district planning decisions partly because the district lacked accurate data to guide priority setting ( Maluka *et al*., 2010b). Epidemiological data was another common formal criterion. Here, priority areas were identified based on the epidemiological data. For example in Malindi district in Kenya, during the district planning process, they used incidence and mortality rates to identify priority areas such as malaria ( Bukachi *et al*., 2014). However, in some instances the use of epidemiological data to guide priority setting was limited by the lack of accurate data. Maluka *et al.* (2010b) found that epidemiological data rarely informed decisions during the district planning process because of inadequate and unreliable data. Historical planning and allocation was also a common formal criterion. Historical planning and allocation mean the current, and subsequent periods priorities are set based on the previous periods priorities. For example, in Mbarali district in Tanzania, historical approach was used to allocate resources across departments during the district planning process ( Maluka *et al*., 2010b). This was attributed to lack of credible evidence and weak information collection and management systems. However, in Plymouth primary care trusts in England, the tendency to “recycle” ideas during operational service improvement priority setting was attributed to starting the priority setting process late hence limited time to develop new priorities ( Goodwin & Frew, 2013).

The informal criteria used in decision making included, political interests, donor interests, regional health managers’ interest or perceptions, and professional experience and expertise. This appeared to be more perverse in LMICs compared to HICs. For example in Tanzania, despite the fact that malaria was the leading cause of morbidity and mortality, a shift in political priority to HIV/AIDs meant that the latter got more allocation of funds ( Maluka *et al*., 2010b). This was thought to be due to, among others, the fact that LMIC settings were characterized by lack of quality data/evidence in the priority settings ( Maluka *et al*., 2010b). In LMICs it also appeared that donor interests significantly influenced priority setting decisions. For example, in Malindi district (Kenya), donor initiatives had to be prioritized and they gave an example of a tetanus campaign not being a priority for the district, but they had to include it in their district annual plan due to the fact that donors wanted to fund it ( Bukachi *et al*., 2014).

### Process of priority setting

**Figure 1.**
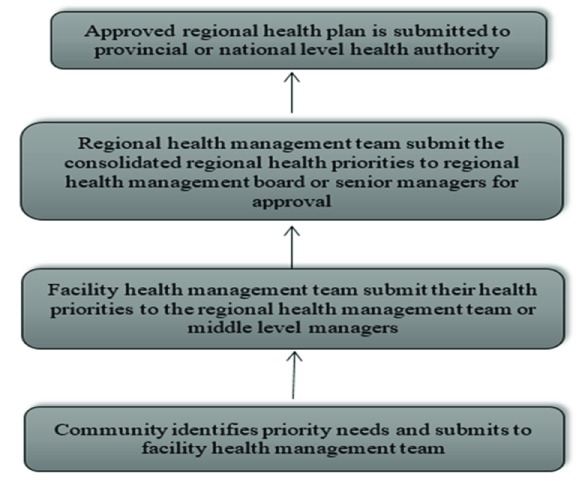
Illustration of the priority setting process at the regional level.

The process of priority setting was examined in the context of the planning and resource allocation process (or budgeting). Variation in the priority setting process was dependent on whether a priority setting framework was used. For instance, when PBMA was used, the process followed the stages involved in the framework. In LMICs where majority did not use priority setting framework, the process followed the decision-making structures. On paper, the process was participatory and began at the community level where they identified their priority health needs and shared with the facility level management through health committees ( Maluka *et al*., 2010b; Maluka, 2011; Nyandieka *et al*., 2015; Zulu *et al*., 2014). The facilities health management teams then compiled their priorities in line with the communities and submitted to the regional health management team at the regional level. The regional health management team then compiled the priorities into a consolidated regional health plan. At this level, the consolidated plans were reviewed and approved by a regional health management board before submission to provincial or national level authority ( Figure 1). However, implementation of the process differed in that participatory planning was rarely achieved ( Maluka *et al*., 2010b; Maluka, 2011). Several studies revealed that the community or the lower level facility managers were not sufficiently involved in the regional priority setting process ( Maluka *et al*., 2010b; Maluka, 2011; Nyandieka *et al*., 2015). Further, regional level priority setting process heavily relied on national guidelines ( Table 3). This meant that in practice, the priority setting process was more of a top-down approach.

### Evaluation of priority setting

Based on the review of empirical literature on evaluation of priority setting, two paradigms can be drawn: consequentialism and proceduralism. Consequentialism holds that the consequences of the process are the ultimate judgment on its success or failure i.e. “the end justifies the means” ( Jan, 2014). Proceduralism is a belief that value is not only derived from the outcomes of a process, but also the process itself. 11 of the 16 studies that evaluated priority setting process reported the use of procedural conditions exclusively in the evaluation of priority setting, three studies reported the incorporation of both procedural and consequential conditions, while two studies reported the evaluation of priority setting processes using consequential conditions alone.

### The procedural measures of priority setting

A common proceduralist framework for evaluating priority setting practices is the accountability for reasonableness (AFR) framework. This framework was reported to have been used in 12 out of the 16 studies that evaluated priority setting processes, with one reporting the combination of AFR and consequential conditions. AFR is a framework that argues that if people cannot agree on principles then they can at least agree on a process that results in decisions that stakeholders can perceive as fair, reasonable and legitimate ( Daniels & Sabin, 1998). It puts emphasis on generating a procedure that ensures fairness and legitimacy rather than having outright principles or values that yield the ‘right answers’( Friedman, 2008). AFR proposes four conditions that are to be met for a fair priority setting process. These conditions are: 1) *relevance-* the rationale for priority setting must be based on relevant reasons (evidence, principles or guidelines) that fair minded people agree are contextually relevant, 2) *Publicity*- priority setting decisions and their rationales must be made available and accessible to all the stakeholders, 3) *Appeal-* there must be a provision to enable the challenging of decisions and revision of decisions when need be, and 4) *Enforcement* - there must be either voluntary or public regulation of the process to ensure that the above three conditions are met. Table 4 outlines the procedural conditions used to evaluate priority setting in the selected studies, and the number of studies that reported compliance with these conditions.

**Table 4.** The procedural conditions used and the number of studies that reported having met the condition.

Procedural Conditions used	Number of studies that met the condition
Use of evidence/information	10/13
Stakeholder engagement	7/13
Relevance	3/12
Publicity	1/12
Appeal/Revisions	1/12
Enforcement	0/12
Data not to be used as a crutch ^*^	0/1
One on one meetings	1/2

* Data not to be used as a crutch means putting less emphasis on having all the ‘data’ to support a decision and more on drawing out opinions from the expert group

### Relevance condition

Of the 12 papers that reported the use of AFR, only three met the relevance condition ( Table 4). Failure to meet this condition was mainly due to difficulties in engagement of a broad range of stakeholders, more so the communities. Two key reasons were identified to be contributing to the poor stakeholder involvement. The first was limited resources (financial and time), which were perceived to be critical for broader stakeholder engagement ( Maluka *et al*., 2010a; Maluka *et al*., 2010b; Maluka, 2011; Maluka *et al*., 2011a). For example, In Mbarali district in Tanzania, health committees that represented community views in the district planning process were non-functional because of lack of funds to pay members for representation ( Maluka *et al*., 2010b). Nyandieka *et al.* (2015) found that in Malindi district in Kenya, only health personnel were involved in priority setting for EMOC services due to lack of time to involve other stakeholders. Second was the perception that the public lacked the knowledge and skills required in priority setting. In Tanzania for example, Maluka *et al.* (2010a), on examination of how to improve district level health planning and priority setting, found that some members the among decision makers felt that the public did not have the capacity to effectively contribute to priority setting decisions, hence their involvement was not helpful.

Another key reason for not meeting the relevance condition was failure to use evidence/ principles/ guidelines as major considerations in priority setting. The use of evidence in priority setting was more prominent in the HICs ( Gibson *et al*., 2006; Goodwin & Frew, 2013; Menon *et al*., 2007; Robinson *et al*., 2012) than the LMICs. This was because priority setting in LMICs was characterized by historical allocations and subjective considerations ( Table 3). For example, In Mbarali district in Tanzania, Maluka *et al.* (2010b) observed that a given year’s priorities were largely based on previous years with minor adjustments for demography or political factors as the district lacked accurate data.

### The publicity condition

Of the 12 papers that reported using AFR, only one met the publicity condition ( Table 4). Majority of the papers attributed the failure to meet this condition to constant failure by decision makers to provide rationale for decisions made ( Bravo Vergel & Ferguson, 2006; Bukachi *et al*., 2014; Gibson *et al*., 2006; Maluka *et al*., 2010a; Zulu *et al*., 2014). For example, Bravo Vergel & Ferguson (2006), observed that though the primary care trusts in England made some effort to communicate priority setting policies through general practitioners and in some few instances through flowcharts and leaflets, the provision of rationale for the decisions were never given. In Mbarali district in Tanzania, there were attempts by district officials to communicate decisions and directives to lower levels through meetings and letters, however, the content of the information did not include rationales ( Maluka *et al*., 2010b). In Kapiri-Mposhi District in Zambia, lack of funds, low literacy levels and lack of public interest and awareness were cited as key reasons in failure to communicate decisions thus not meeting the publicity condition ( Zulu *et al*., 2014).

### The appeals and revisions condition

Of the 12 papers that reported using AFR, only one met the condition ( Table 4). Failure to meet this condition was mainly attributed to lack of an appeals culture and lack of formal mechanisms of appealing decisions. In LMICs, appealing decisions made by higher authorities was perceived to be a tradition that did not exist. For example, a study done in Mbarali district in Tanzania revealed that due to a tradition of not appealing decisions, the public did not know that appealing decisions was an act they could practice ( Maluka *et al*., 2010a). Zulu *et al.* (2014) also found that appealing decisions in Kapiri-Mposhi District in Zambia was very difficult and was not perceived to be an option even by the decision makers to higher authorities. In HICs, the studies revealed limited or lack of formal mechanisms to appeal decisions ( Bravo Vergel & Ferguson, 2006; Gibson *et al*., 2006; Menon *et al*., 2007; Robinson *et al*., 2012). For example, in Calgary health region in Canada, decisions on budget planning were validated through voting and consultations with physicians and managers, however, there were limited opportunities to revisit decisions once made ( Gibson *et al*., 2006). Robinson *et al.* (2012) on examining priority setting and rationing in five PCTs in England, found that they had limited formal processes in place for handling disputes.

### The enforcement condition

Of the 12 papers that reported using AFR, none met the enforcement condition ( Table 4). Failure to enforce the other three conditions was attributed to failure to empower the leadership at the regional levels. The ability of the leaders (community representatives and healthcare managers) was thought to be critical in managing the priority setting processes, more so, in ensuring stakeholder involvement ( Bukachi *et al*., 2014; Maluka *et al*., 2010b; Robinson *et al*., 2012). For example in Mbarali district in Tanzania the committees, boards and even the politicians were liable to oversee the planning and priority setting process thus ensure community values and involvement in the process, however, this was not possible as they lacked the knowledge and skills ( Maluka, 2011). Bukachi *et al.* (2014) on examining the gap in healthcare priority setting in Malindi district in Kenya, found that the leadership at the district level could not enforce all the conditions as there were decisions left to national level managers.

### The outcome measures of priority setting

Outcome measures of priority setting were also used to evaluate the process. These were only used in studies done in HICs. Table 5 outlines the outcome criteria used to evaluate priority setting in selected studies and the number of studies that reported success in meeting these outcome criteria.

**Table 5.** The outcome measures and the number of studies that reported having the condition.

Outcome measures	Number of studies that met the condition
Efficiency/Effectiveness	4/5
Shifted or reallocated resources/ disinvestment of resources	1/4
Options for service delivery redesign	2/3
Improved knowledge of a particular service area	2/2
Evaluation of historical services	2/2
Improved patient outcomes	0/2
Stakeholder satisfaction	1/1
Increased acceptability	1/1
Increased recommendations for use elsewhere	1/1
Budget savings and service improvement	1/1
Stakeholder clarity or understanding	0/1

The most common outcome measures noted in majority of the studies were efficiency, reallocation of resources and options for service delivery redesign. To achieve these, PBMA was used in four studies ( Cornelissen *et al*., 2014; Goodwin & Frew, 2013; Mitton *et al*., 2002; Mitton & Donaldson, 2003) and MCDA together with business templates in the remaining study ( Robinson *et al*., 2012). Most of the papers perceived efficiency as one of the crucial outcomes of a successful priority setting process. For example, in a study done to evaluate the effectiveness of using PBMA in an English primary care trust, it was found that the use of PBMA resulted in technical efficiencies that led to a substantial reduction in hospital activities which was a target for the region ( Goodwin & Frew, 2013). There was a general consensus that the ultimate goal when using PBMA in priority setting was to identify areas for resource reallocation ( Goodwin & Frew, 2013; Mitton *et al*., 2002; Mitton & Donaldson, 2003). However, the studies found that implementing resource reallocation decisions was difficult ( Table 5). Failure to implement proposed decisions was mainly perceived to be due to lack of evidence to support decisions and lack of capacity or authority by members leading the priority setting process to effect actual reallocation of resources. In England, reallocation of resources was also perceived to be a new culture for decision makers. For example, Goodwin & Frew (2013) found that some of the challenges that faced implementation of reallocation of resources in Plymouth primary care trusts in England, were that it represented a major cultural shift and the actors leading the priority setting initiative were not senior enough to be proposing large scale reallocations that had major financial implications. Cornelissen *et al.* (2014) on evaluation of PBMA implementation as a priority setting tool in Central Okanagan in Canada, found that lack of evidence to support investment/disinvestment proposals was a hindrance to resource reallocations. An interesting outcome measure that came up as a slightly more convenient version of resource reallocation is service redesign. Service delivery redesign is an outcome measure that came out due to difficulty in achieving sufficient resource release for resource reallocation. Service delivery redesign is basically offering the same services in a different way with an aim to be more efficient. For example, in chinook health region in Canada, the panel reached a consensus that finding areas to release resources was difficult, therefore, they proposed that chronic management services to be redesigned and services integrated to be efficient ( Mitton *et al*., 2002). This was hoped to lead to better provider relations, even though, resource release wasn’t achieved immediately.

## Discussion

From this review, we highlight a number of observations. First, that empirical literature on evaluation of meso level priority setting is scarce. This is concerning, given that the meso level is a critical component of the healthcare system especially in LMICs. This is because it is the level at which much of the priority setting action in health systems actually takes place. Therefore, without a sound evaluation of the existing priority setting practices, past problems cannot be identified and lessons shared.

Second, the empirical literature reveals that priority setting practices at the meso level is mostly ad hoc, with few studies reporting the use of systematic priority setting processes and frameworks, and all but one were in HICs. Systematic processes and frameworks are meant to guide decision makers in ensuring decisions are consistent, efficient and fair ( Kapiriri & Razavi, 2017). Therefore, their use is important in efforts aimed at achieving health sector goals of efficiency and equity. However, the use of systematic approaches has been shown to be hampered by their complexity and resource requirements ( Kapiriri & Razavi, 2017). Further, the use of systematic approaches is accompanied by evidence based decision making which heavily relies on quantifiable data ( Robinson *et al*., 2012). This perhaps explains why they were not institutionalized in LMICs which suffer greater resource constraints and lack quality data. The fact that the use of systematic approaches was not common, especially in LMICs means that their priority setting processes are ad hoc. This is consistent with priority setting literature in LMICs ( Hipgrave *et al*., 2014; Kapiriri & Martin, 2007; Youngkong *et al*., 2009). However, the success of the local cultural framework in Zambia suggests that systematic approaches can be adapted to suit the context where used. This will ensure that the benefits of systematic approaches are reaped while at the same time considering the available resources.

Third, both formal and informal priority setting criteria were used in decision making, however, informal criteria were mostly used in LMICs. The use of informal criteria in LMICs is not surprising given the lack of systematic priority setting processes and framework. Systematic priority setting processes and frameworks seemed to facilitate the incorporation of formal criteria such as efficiency and equity in priority setting processes, while ad hoc priority setting processes facilitated the use of informal criteria. This is because when confronted with complex decisions and without a guiding framework, decision makers tend to be intuitive or subjective rather than objective. Further, the review established that LMICs lack accurate data that is essential for objectivity in decision making.

Fourth, most of the priority setting processes failed to meet procedural conditions. Procedural conditions are based on deliberative democratic principles aimed at achieving fairness and legitimacy of priority setting processes. Therefore, gaps identified in meeting the procedural conditions provide a basis for improvements in meso level priority setting processes. The relevance condition for example, was not met in most instances because of failure to involve all relevant stakeholders. The exclusion of the communities is particularly concerning given that their involvement in meso-level priority setting is key to reducing health inequities ( Bukachi *et al*., 2014). This is because the involvement of the community amongst other relevant stakeholders ensures that decisions are not dominated by individual interests. The publicity condition was also not met mainly because of failure by decision makers to provide rationale for decisions. The failure to provide rationale for decisions makes the communication of decisions a passive process with no intention to engage the recipients. This creates mistrust between stakeholders and decision makers and they are unlikely to accept the decisions as fair ( Gibson *et al*., 2005). The appeals and revisions condition was also not met because of lack of appeals culture and the lack of formal mechanisms of appealing decisions. This shows that the priority setting processes were inflexible. Further, this could be interpreted as a show of power by the decision makers to avoid scrutiny of decisions made. It is concerning given that through revisions, decision makers are able to improve the quality of decisions as it provides an opportunity to include emerging issues and to correct errors ( Sibbald *et al*., 2009). The enforcement condition was also not met due to failure to empower the leadership at the regional levels. The regional leaders lacked the technical capacity and autonomy to enforce the rules of fairness. This underlies the importance of strengthening leadership as a first step towards improving the fairness of priority setting processes. Regional leaders can be strengthened through management development strategies and increased autonomy in regional priority setting processes.

Finally, outcome measures of priority setting were only used in HICs. This is perhaps due to the use of systematic priority setting processes and frameworks in HICs which led to certain predetermined outcomes which can be used to establish success or failure. For example, marginal analysis in PBMA involves the exploration of options available to reallocate resources. Therefore, reallocation of resources is an expected outcome when the framework is successfully used. However, priority setting processes failed to meet certain outcome criteria. For instance, reallocation of resources was not achieved in most instances. This is worrying given the dynamic nature of healthcare needs ( Barasa *et al*., 2015a). Failure to reallocate resources was mainly attributed to lack of evidence and lack of capacity or authority by members leading the priority setting process to effect actual reallocation of resources. These challenges further highlight the need to strengthen leadership and health information systems. Improved patient outcome was another common outcome criterion that was not met. This is because achieving improved patient outcome is a long-term goal of priority setting processes and frameworks. It rather puts emphasis on the need to examine in greater detail the impact of priority setting processes in the long run.

## Limitations

Our study has several limitations. First, it is likely that studies of priority setting in HIC and LMIC focus on different things making comparison problematic. For instance, it is likely that HIC priority setting studies do not focus on informal processes, while LMIC studies have a special interest in informal process resulting in the impression that informal processes are more common in LMICs. Second, there are very few empirical studies that have evaluated priority setting at the meso-level. Third, there is likelihood that we did not capture all the studies. However, the selection of papers to include in this review was purposive rather than exhaustive because our purpose was interpretive rather than predictive ( Thomas & Harden, 2008). This implies that it was not mandatory to locate every available paper because the findings of our conceptual synthesis would not change if 10 rather than 5 papers comprise of the same concept, but will depend on the range of concepts found in the papers, their context, and whether they agree or not ( Thomas & Harden, 2008).

## Conclusions

Understanding the priority setting experiences of different regions of the world is integral to identifying good practice and areas of improvement in health systems. Priority setting at the meso-level is particularly important, given the health sector reforms towards decentralized health systems. Based on our review, systematic priority setting processes and frameworks are a positive addition towards achieving efficiency and equity in healthcare systems. Further, they are crucial to subduing the influence of informal criteria in decision making. However, there is need to tailor them to suit various context. Our review also reveals that meeting both procedural and outcome measures of priority setting are crucial to improving priority setting practices. We therefore, suggest the integration of both process and outcome measures during priority setting and resource allocation.

## Supplementary material

Supplementary File 1: PRISMA flow diagram.

Supplementary File 2: PRISMA checklist. This checklist was completed with an unpublished version of the manuscript submitted to F1000Research.

## References

[ref-1] Barasa EW Cleary S Molyneux S: Setting healthcare priorities: a description and evaluation of the budgeting and planning process in county hospitals in Kenya. *Health Policy Plan.* 2017;32(3):329–337. 10.1093/heapol/czw132 27679522 PMC5362066

[ref-2] Barasa EW Molyneux S English M: Setting Healthcare Priorities at the Macro and Meso Levels: A Framework for Evaluation. *Int J Health Policy Manag.* 2015a;4(11):719–732. 10.15171/ijhpm.2015.167 26673332 PMC4629697

[ref-3] Barasa EW Molyneux S English M: Setting healthcare priorities in hospitals: a review of empirical studies. *Health Policy Plan.* 2015b;30(3):386–96. 10.1093/heapol/czu010 24604831 PMC4353893

[ref-4] Bravo Vergel Y Ferguson B: Difficult commissioning choices: lessons from English primary care trusts. *J Health Serv Res Policy.* 2006;11(3):150–4. 10.1258/135581906777641749 16824261

[ref-5] Bukachi SA Onyango-Ouma W Siso JM: Healthcare priority setting in Kenya: a gap analysis applying the accountability for reasonableness framework. *Int J Health Plann Manage.* 2014;29(4):342–361. 10.1002/hpm.2197 23775594

[ref-6] CASP UK: Critical Appraisal skills programme (CASP) Check lists [Online].2017; Accessed 24th November 2017. Reference Source

[ref-7] Cornelissen E Mitton C Davidson A: Changing priority setting practice: the role of implementation in practice change. *Health Policy.* 2014;117(2):266–274. 10.1016/j.healthpol.2014.04.010 24815208

[ref-8] Daniels N Sabin J: The ethics of accountability in managed care reform. *Health Aff (Millwood).* 1998;17(5):50–64. 10.1377/hlthaff.17.5.50 9769571

[ref-9] Friedman A: Beyond accountability for reasonableness. *Bioethics.* 2008;22(2):101–12. 10.1111/j.1467-8519.2007.00605.x 18251770

[ref-10] Gibson J Mitton C Martin D: Ethics and economics: does programme budgeting and marginal analysis contribute to fair priority setting? *J Health Serv Res Policy.* 2006;11(1):32–37. 10.1258/135581906775094280 16378530

[ref-11] Gibson JL Martin DK Singer PA: Evidence, economics and ethics: resource allocation in health services organizations. *Healthc Q.* 2005;8(2):50–9,4. 10.12927/hcq..17099 15828568

[ref-12] Goodwin E Frew EJ: Using programme budgeting and marginal analysis (PBMA) to set priorities: reflections from a qualitative assessment in an English Primary Care Trust. *Soc Sci Med.* 2013;98:162–8. 10.1016/j.socscimed.2013.09.020 24331895

[ref-13] Hannes K: Chapter 4: Critical appraisal of qualitative research. In: Noyes J, Booth A, Hannes K, Harden A, Harris J, Lewin S, Lockwood C (ed.) *Supplementary Guidance for Inclusion of Qualitative Research in Cochrane Systematic Reviews of Interventions* 2011 Reference Source

[ref-14] Hipgrave DB Alderman KB Anderson I: Health sector priority setting at meso-level in lower and middle income countries: lessons learned, available options and suggested steps. *Soc Sci Med.* 2014;102:190–200. 10.1016/j.socscimed.2013.11.056 24565157

[ref-15] Jan S: Proceduralism and its role in economic evaluation and priority setting in health. *Soc Sci Med.* 2014;108:257–61. 10.1016/j.socscimed.2014.01.029 24647102

[ref-16] Kapiriri L Martin DK: A strategy to improve priority setting in developing countries. *Health Care Anal.* 2007;15(3):159–167. 10.1007/s10728-006-0037-1 17922194

[ref-17] Kapiriri L Norheim OF Martin DK: Priority setting at the micro-, meso- and macro-levels in Canada, Norway and Uganda. *Health Policy.* 2007;82(1):78–94. 10.1016/j.healthpol.2006.09.001 17034898

[ref-18] Kapiriri L Razavi D: How have systematic priority setting approaches influenced policy making? A synthesis of the current literature. *Health Policy.* 2017;121(9):937–946. 10.1016/j.healthpol.2017.07.003 28734682

[ref-19] Maluka S Kamuzora P San Sebastián M: Improving district level health planning and priority setting in Tanzania through implementing accountability for reasonableness framework: Perceptions of stakeholders. *BMC Health Serv Res.* 2010a;10:322. 10.1186/1472-6963-10-322 21122123 PMC3009977

[ref-20] Maluka S Kamuzora P San Sebastiån M: Decentralized health care priority-setting in Tanzania: evaluating against the accountability for reasonableness framework. *Soc Sci Med.* 2010b;71(4):751–759. 10.1016/j.socscimed.2010.04.035 20554365

[ref-21] Maluka S Kamuzora P Sansebastian M: Implementing accountability for reasonableness framework at district level in Tanzania: a realist evaluation. *Implement Sci.* 2011a;6:11. 10.1186/1748-5908-6-11 21310021 PMC3041695

[ref-22] Maluka SO: Strengthening fairness, transparency and accountability in health care priority setting at district level in Tanzania. *Glob Health Action.* 2011;4. 10.3402/gha.v4i0.7829 22072991 PMC3211296

[ref-23] Maluka SO Hurtig AK Sebastian MS: Decentralization and health care prioritization process in Tanzania: from national rhetoric to local reality. *Int J Health Plann Manage.* 2011b;26(2):e102–120. 10.1002/hpm.1048 20603818

[ref-24] Mckneally MF Dickens BM Meslin EM: Bioethics for clinicians: 13. Resource allocation. *CMAJ.* 1997;157(2):163–7. 9238146 PMC1227741

[ref-25] Menon D Stafinski T Martin D: Priority-setting for healthcare: who, how, and is it fair? *Health policy.* 2007;84(2–3):220–233. 10.1016/j.healthpol.2007.05.009 17628202

[ref-26] Ministry of Health: National and county health budget analysis FY 2016/17. Nairobi.2017 Reference Source

[ref-27] Mitton C Donaldson C: Health care priority setting: principles, practice and challenges. *Cost Eff Resour Alloc.* 2004;2(1):3. 10.1186/1478-7547-2-3 15104792 PMC411060

[ref-28] Mitton C Donaldson C Halma L: Setting Priorities and Allocating Resources in Regional Health Authorities: A Report from Two Pilot Exercises Using Program Budgeting and Marginal Analysis. *Healthc Manage Forum.* 2002;15:1–9. 10.1016/S0840-4704(10)60200-1

[ref-29] Mitton CR Donaldson C: Setting priorities and allocating resources in health regions: lessons from a project evaluating program budgeting and marginal analysis (PBMA). *Health Policy.* 2003;64(3):335–48. 10.1016/S0168-8510(02)00198-7 12745172

[ref-30] Nyandieka LN Kombe Y Ng'ang'a Z: An assessment of priority setting process and its implication on availability of emergency obstetric care services in Malindi district, Kenya. *Pan Afr Med J.* 2015;22:156. 10.11604/pamj.2015.22.156.7296 26889337 PMC4742024

[ref-31] Robinson S Williams I Dickinson H: Priority-setting and rationing in healthcare: evidence from the English experience. *Soc Sci Med.* 2012;75(12):2386–93. 10.1016/j.socscimed.2012.09.014 23083894

[ref-32] Shamseer L Moher D Clarke M: Preferred reporting items for systematic review and meta-analysis protocols (PRISMA-P) 2015: elaboration and explanation. *BMJ.* 2015;350:g7647. 10.1136/bmj.g7647 25555855

[ref-33] Sibbald SL Singer PA Upshur R: Priority setting: what constitutes success? A conceptual framework for successful priority setting. *BMC Health Serv Res.* 2009;9:43. 10.1186/1472-6963-9-43 19265518 PMC2655292

[ref-34] Thomas J Harden A: Methods for the thematic synthesis of qualitative research in systematic reviews. *BMC Med Res Methodol.* 2008;8:45. 10.1186/1471-2288-8-45 18616818 PMC2478656

[ref-35] Tuba M Sandoy IF Bloch P: Fairness and legitimacy of decisions during delivery of malaria services and ITN interventions in Zambia. *Malar J.* 2010;9:309. 10.1186/1475-2875-9-309 21040552 PMC2988042

[ref-36] Youngkong S KapirirI L Baltussen R: Setting priorities for health interventions in developing countries: a review of empirical studies. *Trop Med Int Health.* 2009;14(8):930–939. 10.1111/j.1365-3156.2009.02311.x 19563479

[ref-37] Zulu JM Michelo C Msoni C: Increased fairness in priority setting processes within the health sector: the case of Kapiri-Mposhi District, Zambia. *BMC Health Serv Res.* 2014;14:75. 10.1186/1472-6963-14-75 24548767 PMC3932790

