# Supplementary figures and images for: Evaluating healthcare priority setting at the meso level: A thematic review of empirical literature
Source: Wellcome Open Res. 2018 Feb 20;3:2. Originally published 2018 Jan 8. [Version 2] doi: 10.12688/wellcomeopenres.13393.2 (PMC5814743; doi:10.12688/wellcomeopenres.13393.2)

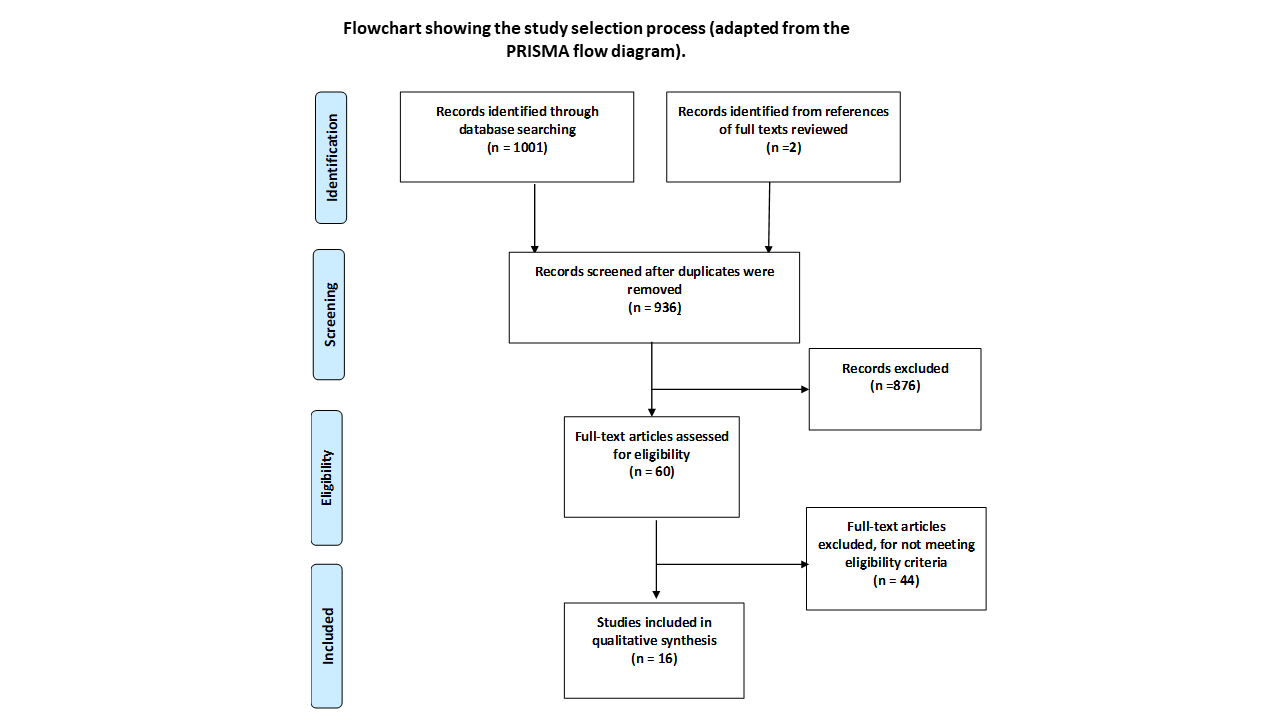

Supplement: Supplementary file 1 [file wellcomeopenres-3-15287-s0000.tgz › 394de424-89ca-4369-a164-ac4f12d60cca.tif]
