# Supplementary material for: Evaluating healthcare priority setting at the meso level: A thematic review of empirical literature
Source: Wellcome Open Res. 2018 Feb 20;3:2. Originally published 2018 Jan 8. [Version 2] doi: 10.12688/wellcomeopenres.13393.2 (PMC5814743; doi:10.12688/wellcomeopenres.13393.2)
Supplement: Peer review history

# Peer review: Referee response for version 1

**DOI:** 10.21956/wellcomeopenres.14541.r30237

**Published:** 2018-02-14

**Authors:** Amani Thomas Mori

**Affiliations:** 1 University of Bergen, Bergen, Norway

The paper by Dennis *et al*. evaluates healthcare priority-setting at meso level, and shows that the few available studies are mainly from LMIC countries and the processes are mostly top-down and unsystematic. The study generates a good summary and open-up gaps for more research. The paper can be improved in a few sections that are described below:

1. In the methods section, the authors should give rationales for exclusion criteria e.g. why was the search limited to between 1997 and 2017?
2. In the methods section, the authors should also mention in the paper who did the search and extraction of the data.
3. Given that table 2 reports quality appraisal, one could easily think the numbers under the columns labelled  “yes, somewhat and no/not clear” are the scores rather than #of papers. It should be properly labelled to show that the # reflect number of papers.
4. In the results section, page 9, under Relevance criteria it is written: “ Of the 12 papers that used AFR, only 3 met the relevance criteria (Table 4)”. The authors need to use passive voice in most cases to show that they report what the studies reported. For example instead of saying 3 met the relevance criteria….they should say “3 reported that the relevance criteria was met…. The same can be said of the Title of Table 4, instead of “…number of studies that met the conditions” they should write  “….number of studies which reported that the conditions were met”. Make necessary changes in the corresponding text from page 9-10 if you agree with my observation.

I have read this submission. I believe that I have an appropriate level of expertise to confirm that it is of an acceptable scientific standard.

# Peer review: Referee response for version 1

**DOI:** 10.21956/wellcomeopenres.14541.r30236

**Published:** 2018-02-14

**Authors:** Nicola Foster

**Affiliations:** 1 Health Economics Unit, School of Public Health and Family Medicine, University of Cape Town, Cape Town, South Africa

The work presented, fills an important gap in the literature related to meso-level decision-making. The authors rightly argue that this is an important area where implicit priority-setting happens regularly - but is under studied. My recommendation is that the paper should be indexed, and my comments below are meant to merely strengthen some of the analyses or discussion; and hopefully to provide some food for thought. Comments are organised as either major or minor comments.

**Major comments**

- In methods/ results (page 3): would suggest moving the first paragraph under "quality appraisal" to the methods section rather and you may want to expand on this a bit more as a key component of your analysis. Likewise, in the results section, it would be good to see more of a description of what you found in the quality appraisal i.e. speaking a bit more to Table 2. In particular, it may be good to also speak a bit about why four of the studies did not score well in terms of how the data analysis was conducted.
- In thinking about the results section of the paper and subsequent discussion of the results, it may be helpful to include a bit more information on the main findings of each of the studies in Table 1 - this would then allow the reader to better evaluate the written section from page 6 onwards. 
- Under the results and linked to the discussion, the findings related to processes for disinvestment is interesting and my sense is that it is a key challenge in all health systems. In the results and discussion, it may be useful to be a bit clearer about the distinction between process and institutions. So, for example, while not explicitly discussed, the lack of institutions seem to be important on page 10, sub-heading the enforcement condition; and is tangentially discussed in the discussion section in the second paragraph, the fourth paragraph and the 5th paragraph on page 12. It may strengthen the results and discussion section by making the link between the role of institutions as it comes up in the results more explicit.
- The finding mentioned in the discussion (3rd paragraph), that informal priority-setting criteria were more common in LMIC compared to HIC settings was not immediately clear to me from the results section of the paper.
- The paper could also be strengthened by adding a figure to show the relationship between codes in the coding framework (mentioned in the methods section under data extraction).
- In your discussion, it may also be useful to reflect a bit more on how the study limitations especially related to which priority-setting processes are reported and studied on, may influence you results. For example, is it possible that there may a reporting bias in the finding related to more informal priority-setting  criteria in LMIC compared to HIC settings - in that could it be that it is more likely that informal priority-setting process would be *described/ studied* in LMIC settings? While in HIC there may be less interest in studying/ reporting on informal priority-setting processes in academic literature?
- The discussion could be strengthened by making additional suggestions on possible questions that future research may be designed to address.

 

**Suggested minor edits**

- Methods < eligibility criteria (page 3): may be helpful to here remind the reader again as to the definition of meso-level used in this study.
- In general, one want to refrain from using numerals if the value is under ten, but to rather write these out in words. Particularly on page 6, under "frameworks of priority-setting".
- Possible type on page 3, section results, line 2: lead to led?

I have read this submission. I believe that I have an appropriate level of expertise to confirm that it is of an acceptable scientific standard.

# Peer review: Referee response for version 1

**DOI:** 10.21956/wellcomeopenres.14541.r29588

**Published:** 2018-01-23

**Authors:** Shakira Choonara

**Affiliations:** 1 SRHR Africa Trust, Johannesburg, South Africa

This article should be accepted for indexing given its identification of a clear gap within empirical research around priority setting in health systems. The paper further adds to the body of health systems knowledge and research which highlights the importance and value of qualitative methods in systematic review processes. The paper provides an interesting read and comparison between developed and developing settings. Overall the objective of the review followed through by concise methods adopted, analysis and conclusions offer an interesting contribution to the literature, although minor suggestions must be considered by authors prior to publication, with the aim strengthening the write-up, policy relevance and possibly follow-up publications;

- The abstract should state in the background that the review is conducted globally (developed and developing countries) to reflect the objective of the paper. The abstract assumes the reader understand the categorization “meso” level of the health system and that it is neglected. It may be useful to define the meso level as in the introduction.
- In the introduction section, a few more studies could be cited besides the United Kingdom and Kenya as it is not clear to the reader why those two examples were selected, the connection or what the implication of mention is.
- The section “The priority setting activity that was studied across all the selected papers was the planning and resource allocation (or budgeting) process at the regional level” could be emphasised in the background to explain the definition/ components of priority setting and included in the abstract.
- It is recommended that some of the sections under results be moved to the methods section e.g. the first paragraph – synthesis of selected papers and use of the Critical Appraisal Skills Programme (CASP) tool.
- The methods section could be strengthened by stating the inclusion and exclusion criteria more explicitly.
- The discussion section of the paper is particularly well-structured, although both in the introduction and discussion the importance of different contexts from a range of factors example, resources to political systems should be highlighted and unpacked further. In fact, it would be beneficial to the reader summarizing the results section by contrasting high and low income findings through a table versus the text. Alternatively, the comparison could serve as the focus of a follow-up publication.
- The conclusion both in the abstract and the paper could be more useful if it is extended or clearly states what policy-makers/ key decision-makers should consider in priority setting processes – i.e. the paper present the opportunity to moving beyond summarising the literature, with policy/ relevance for practice.

I have read this submission. I believe that I have an appropriate level of expertise to confirm that it is of an acceptable scientific standard.
